# Analysis of Some Calculi Extracted from the Bladder of a Dog

**Published:** 1809-05

**Authors:** M. Fourcroy


### *Analysis of some Calculi extracted from the Bladder of a Dog.* By M. Fourcroy.

In former papers in the Annales de Chimie, I have spoken of calculi or bezoards in the bladders, or other parts of the anatomy of animals. I remarked, that having had occasion to analyse some urinary calculi of the horse, the ox, the sow, and the rat, I discovered them to be formed of carbonate of lime; while the intestinal calculi of the same animals were constantly formed of ammoniacal magnesian phosphate. I have drawn from this striking difference between human calculous concretions and those of animals, a similar conclusion with that from the comparative analysis of the urine of man and of the mammiferæ. I have shewn, that the urine of the latter, not containing any phosphates, these salts transpire in their exudations, and collecting on their nails or hair, are afterwards carried into the intestines.

But, when I published these important observations upon the animal œconomy, I had only examined the calculi of herbivorous animals. I was anxious to examine the urinary and intestinal calculi of carnivorous animals; and the difficulty I experienced in procuring them, made me of opinion, that calculi were much rarer in carnivorous than in herbivorous; for after seven years inquiries, I had not procured one.

This opportunity, however, has since presented itself. M. Dufresne, who omits no opportunity of promoting science, having had occasion to make a preparation from the dead body of a dog, was astonished to find the urinary bladder filled with calculi, arranged together like pieces of mosaic. We have frequently seen the same thing in the human bladder, and still more frequently in the gall bladder. When M. Dufresne brought in the above calculi, I immediately submitted them to analysis, assisted by M. Laugier, whose talents and accuracy are so well known. Having been witness to my numerous analyses of calculi and of bezoards, and guided by the plan, which he has seen us follow, M. Laugier examined with the requisite minuteness, this new description of calculi; while I directed his experiments, and observed the phenomena.

The stones in question were fifty-seven in number, and filled the whole cavity of the bladder. Some were round, and of the size of the end of one's finger; most of them much smaller, presenting three worn faces and a tetrahedral form; the whole were glossy on their surface, and of a white colour slightly yellow. These fragile concretions, almost polished externally, are formed of concentric layers, which are easily separated; their interior is covered with small brilliant crystals; they have in their centre a moveable nucleus of the same nature with the concentric layers.

The animal in which these calculi were found, was a bitch of seven years of age. She had been several times in season, but every possible care had been taken to keep her from dogs; and, we were assured, that she never had been covered. It was also remarked, that in spite of her disease, she was very fat, and she had been fed upon a variety of sweet food.

1. Several of these calculi were reduced into powder, and triturated with caustic potash; when a great quantity of ammonia was extricated.

2. When treated with the blow pipe, the same extrication took place; there was besides developed, a smell of burnt animal matter; after the formation of the charcoal, the dust became white and soft; phosphoric light was emitted, and we at last obtained a transparent globule, which became opaque upon cooling.

3. These entire calculi placed in the muriatic acid were dissolved extremely well, and without any effervescence. They left after their solution a membranous animal substance, which is also presented in all white calculi of whatever nature they are.

4. Upon pouring into this solution oxalate of ammonia, a trifling precipitate, which had all the properties of oxalate of lime.

5. Ammonia poured into the same solution, from which the first precipitate of oxalate of lime had been separated, produced a new voluminous and fleshy precipitate, white and opaque; which was easily re-dissolved by the addition of some drops of sulphuric acid, and the magnesian nature of which was very manifest.

6. The calculi bruised in water are partly dissolved by ebullition, and this solution gave all the appearances of the presence of ammoniacal magnesian phosphate.

These six experiments prove, very exactly, that the calculi in the bladder of a dog are formed of a great quantity of ammoniacal magnesian phosphat, of a small portion of phosphate of lime, and of a membranous like animal matter.

Here

Here then we have in a carnivorous animal, concretions very different from those of herbivorous animals, and similar, in some respects, to some of the calculi in the human bladder. There are earthy phosphates deposited from the urine of quadrupeds, as well as men.

This fact proves, that the kind of food influences both the nature of the humours, and the nature of the matters which are separated from them. Instead of destroying, or even weakening the preceding assertions, with respect to the calculi of herbivorous animals, very different from those of men, this new result must confirm them, by creating a comparison between the phenomena which take place in the human body, with those which take place in the bodies of those mammiferæ, who live upon the same food with mankind.

It still remains to analyse some calculi in the bladders of various kinds of carnivorous animals, in order to obtain a more conclusive result, which I have already done with respect to herbivorous animals, four species of which have furnished urinary concretions. To this ought to be added, the analysis of the intestinal concretions of the same carnivorous animals. Here the difficulty will be great, because anatomical experience shews, that these animals are much less subject than herbivorous to intestinal concretions. All these results, however, must be the fruit of much study and labour. In the mean time, the scientific world may be assured, that no opportunity of examining these subjects will be lost, so far as the French Museum is concerned; and, it is to be hoped, that the same vigilance will be displayed by other naturalists, when occupied with the dissection and preparation of animal subjects.

